# Proteomic comparison of epidemic Australian *Bordetella pertussis* biofilm cells

**DOI:** 10.1128/spectrum.01715-25

**Published:** 2025-09-30

**Authors:** Hiroki Suyama, Laurence Don Wai Luu, Ling Zhong, Mark J. Raftery, Ruiting Lan

**Affiliations:** 1 School of Biotechnology and Biomolecular Sciences, University of New South Wales 7800, Sydney, New South Wales, Australia; 2 Bioanalytical Mass Spectrometry Facility, University of New South Wales 7800, Sydney, New South Wales, Australia; LSU Health Shreveport, Shreveport, Louisiana, USA

**Keywords:** whooping cough, proteomics, pertussis, biofilm, *Bordetella pertussis*, multiple reaction monitoring (MRM), tandem mass tagging (TMT), gene expression, vaccines, PetABC, confocal microscopy, *Bordetella*

## Abstract

*Bordetella pertussis* causes whooping cough, a severe respiratory infectious disease. Studies have compared the currently dominant single nucleotide polymorphism (SNP) cluster I (pertussis toxin promoter allele, *ptxP3*) and previously dominant SNP cluster II (*ptxP1*) strains as planktonic cells. Since biofilm formation is linked with *B. pertussis* pathogenesis *in vivo*, this study compared the biofilm formation capabilities of representative strains of cluster I and cluster II. Confocal laser scanning microscopy found that the cluster I strain had a denser biofilm structure compared to the cluster II strain. Differences in protein abundance of the biofilm cells were then compared using tandem mass tagging and high-resolution multiple reaction monitoring. In total, 1,453 proteins were identified, of which 40 proteins had significant differential abundance between the two strains in biofilm conditions. Of particular interest was a large increase in the abundance of energy metabolism proteins (cytochrome proteins PetABC and BP3650) in the cluster I strain. When the abundance of these proteins was compared between six additional strains from each cluster, it was found that the protein abundance varied between all strains. These findings suggest that there are large levels of individual proteomic diversity between *B. pertussis* strains in biofilm conditions despite the highly conserved genome of the species. Overall, this study revealed visual differences in biofilm structure between *B. pertussis* strains and highlighted strain-specific variation in protein abundance that dominates potential cluster-specific changes that may be linked with the dominance of cluster I strains.

**IMPORTANCE:**

*Bordetella pertussis* causes whooping cough. The currently circulating cluster I strains have taken over previously dominant cluster II strains. It is important to understand the reasons behind this evolution to develop new strategies against the pathogen. Recent studies have shown that *B. pertussis* can form biofilms during infection. This study compared the biofilm formation capabilities of a cluster I and a cluster II strain and identified visual differences in the biofilms. The protein abundance between these strains grown in biofilms was compared, and proteins identified with varied abundance were measured with additional strains from each cluster. It was found that despite the highly conserved genetics of the species, there was varied protein abundance between the additional strains. This study highlights that strain-specific variation in protein abundance during biofilm conditions may dominate the cluster-specific changes that may be linked to the dominance of cluster I strains.

## INTRODUCTION

Whooping cough (pertussis) is a severe respiratory infection caused by the bacterium, *Bordetella pertussis*. Despite widespread global vaccination, a resurgence in pertussis cases has been observed globally (1–3). A contributing factor for the resurgence is the evolution of strains with greater fitness and mismatches with the vaccine allele types (3, 4). In Australia, a whole cell vaccine was introduced in the 1950s, but due to its reactogenicity, it was subsequently replaced by the acellular vaccine (ACV) in 2000 (5). *B. pertussis* is a homogeneous species with a low level of genetic diversity in terms of single nucleotide polymorphisms (SNPs) (6–8). Previous genotyping studies divided the global *B. pertussis* population into 6 SNP clusters (I–VI) based on 65 SNPs, which were further divided into 42 SNP profiles (7). Our studies have shown that strains belonging to SNP cluster I containing the pertussis toxin promoter *ptxP3* allele have overtaken the previously dominant SNP cluster II strains containing the pertussis toxin promoter *ptxP1* allele (7). Further whole-genome sequencing of SNP cluster I strains divided this cluster into six “epidemic lineages” (EL 1–6), which were responsible for the 2008–2012 and 2014–2017 Australian epidemics (9). Interestingly, studies using a mouse model showed that the cluster I strains had greater fitness compared to the cluster II strains regardless of the immunization status of the host, signifying that there are potentially further changes other than antigenic mismatches with the ACV that allow for the increased fitness of the cluster I strains (10).

Transcriptomic studies have shown that there were a large number of genes with altered expression between *ptxP3* and *ptxP1* strains (4). Major changes in virulence factors, including an increase in pertussis toxin production, were identified and hypothesized to increase colonization in mice (4, 11). Furthermore, proteomic studies have identified other changes that have occurred between SNP cluster I and SNP cluster II strains (12–14). Changes in the abundance of proteins associated with immune evasion, virulence, and metabolism have been identified in planktonic cells and their secretome (13). Of key importance, it was seen that there was a decrease in the secretion of type 3 secretion system proteins in cluster I strains, which may be related to a change from type 3 secretion system allele *bscI1* to *bscI3* (9, 12, 13). These additional changes may be associated with the increased fitness observed in the cluster I strains.

Recent studies have shown that biofilms play a key role in *B. pertussis* pathogenesis (15, 16). Biofilms are a community of cells that are encased within a matrix, and cells within biofilms have been shown to be more resilient against antimicrobials and environmental stress (17–19). *B. pertussis* has been shown to form biofilms *in vivo* in the upper respiratory tract of a mouse model, and strains that lack the ability to form biofilms were less effective at colonization (15, 20, 21). Furthermore, increased biofilm formation capability has been linked to increased colonization ability (20). Clinical *B. pertussis* isolates have been shown to form increased biofilm compared to the Tohama I reference strain isolated in the 1950s (17, 22, 23). These studies have demonstrated that biofilms are an important aspect of *B. pertussis* pathogenesis.

Previous studies have identified expression differences between cluster I *ptxP3* and cluster II *ptxP1* strains under planktonic conditions (4, 11–13, 24). However, cells in biofilms are known to be phenotypically distinct from their planktonic counterpart with major changes in transcriptomic and proteomic profiles (17, 23, 25–29). Therefore, comparison between the cluster I and cluster II strains in biofilm conditions may provide additional insights into the adaptation of cluster I strains and the re-emergence of *B. pertussis*. This study examined biofilm-forming capabilities and proteomic changes between cluster I and cluster II biofilms to identify variations that may help explain the increased fitness of the cluster I strains.

## MATERIALS AND METHODS

### Strains and growth conditions

Strains used in this study are listed in Table 1. All strains in this study were pertactin positive, as we wanted to compare whether current cluster I strains have increased fitness due to biofilm differences that were independent of pertactin inactivation. Clinical isolates, L1423 and L1191, were used as representatives of SNP cluster I and SNP cluster II, respectively. Both strains have been previously sequenced and used for comparative infection in a mouse model and proteomic studies in planktonic conditions (10, 13). The additional strains used to confirm changes in the clusters are all from different years but classified as the same SNP profile (SP) as defined by Octavia et al. (7). All cluster I strains are SP13 from the 2008–2010 outbreak in Australia, while all strains in cluster II are SP39 ranging from 2000 to 2006, except for L1191 (Table 1). L1191 was isolated during the same 2008–2010 epidemic period as the cluster I strains, while the additional cluster II strains were isolated from pre-epidemic periods, as the occurrence of epidemic cluster II strains has been rare since 2008.

**TABLE 1:** Strains used in this study

Strain	Cluster	SNP profile	Year	Epidemic lineage^*b*^
L1423^*a*^	I	SP13	2010	EL4
L1397	I	SP13	2010	EL2
L1216	I	SP13	2009	EL3
L1042	I	SP13	2008	EL4
L1398	I	SP13	2010	EL6
L1217	I	SP13	2009	EL4
L1041	I	SP13	2008	EL3
L1191^*a*^	II	SP37	2009	
L479	II	SP39	2000	
L488	II	SP39	2001	
L700^*b*^	II	SP39	2002	
L697	II	SP39	2002	
L517^*b*^	II	SP39	2006	
L504^*b*^	II	SP39	2004	

^*a*^ Denotes strains used in representative tandem mass tagging proteomics.

^*b*^ Epidemic lineage assignment of cluster I strains from Xu et al. (9).

*B. pertussis* strains were grown on Bordet-Gengou (BD Scientific) agar for 3–5 days at 37°C. For liquid culture, a loopful of pure Bvg^+^ colonies was suspended in 20 mL of THIJS media (30) supplemented with heptakis [(2,6-O-dimethyl) β-cyclodextrin] and 1% THIJS supplement and grown for 24 h shaking at 180 rpm at 37°C.

### Confocal laser scanning microscopy

Confocal laser scanning microscopy (CLSM) was used to identify structural changes that occur over time and to identify biofilm maturation. Microscopy was performed on the two representative strains from clusters I (L1423) and II (L1191). A previously established protocol was used with minor changes (31). Briefly, the liquid culture described above was adjusted to an OD_600_ of 0.1/mL and seeded into 24-well polystyrene tissue culture plates (Corning) with 12 mm circular sterile glass coverslips (Livingstone, Thickness: No. 1) on the base. The plate was incubated at a 45° angle so the liquid to air interface was localized on the coverslip. The plate was incubated statically for 5 h to allow attachment of cells before the medium was refreshed (25, 26). The plates were further incubated at 37°C for 24, 48, 72, and 96 h with mild agitation at 60 rpm (32). At each time point, the wells were washed three times with 1× PBS, and the coverslip was carefully removed from the well. The biofilm was fixed with 4% paraformaldehyde and stained with SYTO 9 fluorescent dye (Thermo Fisher Scientific). Z-stack images were captured on the Olympus FluoView FV1200 confocal microscope. Three fields of view were randomly selected per sample. Each field of view covering a surface area of 84.48 × 84.48 µm was imaged with Z-stacks (0.42 µm/slice). All images were imaged at 60× objective (oil immersion, NA 1.35; Olympus). The COMSTAT2 (version 2.1) plugin on ImageJ (version 2.8.0) was used to calculate biomass, surface area, and maximum thickness of biofilms (33). Three biological replicates per strain per time point were performed.

### Biofilm protein extraction

A previously established method for protein extraction from *B. pertussis* cells (13, 27) was used with minor changes. Briefly, all wells of a polystyrene 24-well tissue culture-treated plate (Corning) were seeded with 24-h liquid culture as described above. Each plate represented a single biological replicate, and seven biological replicates were performed per strain. The plates were incubated statically for 5 h at 37°C to allow cell attachment before the medium was refreshed. The plates were then incubated for an additional 96 h, shaking at 60 rpm. At 96 h, the supernatant was removed, and the wells were washed three times with sterile 1× PBS. Disruption buffer (50 mM Tris-HCl, 0.4 mM phenylmethylsulfonyl fluoride, and 2 mM EDTA) was added to inactivate proteases. The plates were water bath ultrasonicated at 37 kHz for 2 min to detach the biofilm cells. All the wells from each individual plate were pooled, centrifuged, and resuspended in disruption buffer. The samples were then probe sonicated, and proteins were extracted using a method previously described by Luu et al. (13, 27). A Qubit Fluorometer (Thermo Fisher Scientific) was used to quantify the protein concentration.

### Protein digestion and TMT labeling

For tandem mass tagging (TMT), 100 µg of protein from each replicate was reduced with dithiothreitol, alkylated with iodoacetamide, and trypsin digested as previously described by Luu et al*.* (34). Samples were cleaned up using Empore high-performance extraction disk cartridges (3M). Following the clean up, the samples were labeled using the TMT10plex and TMT6plex labels as described in the manufacturer’s protocol (Thermo Fisher Scientific). To account for seven biological replicates per strain, a 10plex and 6plex kit was used separately. A pooled reference with all samples was included as a reference for normalization in both experiments. After labeling the peptides with TMT, a styrene divinylbenzene stage tip was used to clean up excess tags. The samples were dried using a Savant SpeedVac (Thermo Fisher Scientific). Samples were resuspended in 0.1% formic acid, then loaded onto the QExactive Orbitrap mass spectrometer coupled with an UltiMate 3000 high-performance nano liquid chromatography system (Thermo Fisher Scientific). Peptides were eluted over a 240-min gradient, ramping from H_2_O:CH_3_CN (98:2, 0.1% formic acid) to H_2_O:CH_3_CN (64:36, 0.1% formic acid) and introduced directly with electrospray ionization with a flow rate of 200 nL/min. Mass spectrometry (MS) spectra were obtained across the mass range of 350–1,750 *m/z* at a resolution of 70,000 *m/z*, and MS/MS resolution at 45,000 *m/z*. The 15 most abundant ions were selected for fragmentation with higher-energy collision dissociation. Two technical replicates were performed for each biological replicate.

### Peptide identification and quantification

The TMT10 and TMT6 experiments were combined and normalized to the pooled reference sample. The MS spectra were imported into the Proteome Discover (Thermo Fisher Scientific) software (version 2.3) and searched using SEQUEST HT against a custom *B. pertussis* database containing complete genomes of Tohama I, CS, B1917 (*ptxP3*), and B1920 (*ptxP1*). Peptide tolerance was set at 4 ppm, and the MS/MS tolerance at 0.04 Da. Variable modifications were set as carbamidomethyl (C) and oxidation (M), and fixed modifications as TMT-6plex (K), TMT-6plex (N-term), TMT-10plex (K), and TMT-10plex (N-term). Enzyme specificity was set as trypsin with a maximum of 1 missed cleavage, and minimum peptides per protein was set as 2. Functional categories were assigned based on Bart et al. (2). pSORTb (version 3.0.2) was used to predict subcellular locations of each protein.

### MRM-hr

To confirm the changes in protein abundance identified in the TMT-MS, high-resolution multiple reaction monitoring (MRM-hr) was used. MRM-hr experiments were run on the two representative strains (L1423 and L1191) and then further performed on six additional strains from each cluster. Biofilms were grown as described for TMT experiments. Control proteins were selected as internal standards to normalize the intensities. These proteins were selected based on an FC = 1 ± 0.1 in the TMT experiment. The analysis was performed on the QExactive Orbitrap mass spectrometer. The analysis was designed using Skyline (version 21.1.0.278). Protein peptides were screened for suitability for MRM-hr. Peptides with potential ragged ends, missed cleavages, or amino acids susceptible to variable modifications were excluded from the analysis. Proteins were excluded unless there was a minimum of two suitable peptides identified. The highest peak height and area were selected for each peptide based on the Skyline automated peak detection method (35). Six biological replicates were performed for the representative strains, while each additional cluster strain was considered a biological replicate for the cluster. Two technical replicates per biological replicate were injected into the mass spectrometer for analysis.

Ten micrograms of protein was trypsin digested as described above. Samples were cleaned up using C18 StageTips (Thermo Fisher Scientific) and injected into the mass spectrometer over a 30-min gradient. The MRM-hr acquisition consisted of a 50 ms MS scan followed by MS/MS scans of 35 candidate ions per cycle (110 ms accumulation time, 35,000 *m/z* resolution). The collision energy for each peptide was determined using Skyline and is available in Table S1. Retention times were scheduled based on pilot non-scheduled MRM-hr runs (36).

### Genome comparison of L1423 and L1191

Complete genomes of *B. pertussis* strains, L1423 (SNP cluster I) and L1191 (SNP cluster II), have previously been sequenced (10). The genome sequences were compared using Mauve (version 20150226) (37). Protein sequences were compared using Clustal Omega (38).

### Statistical analysis

For confocal microscopy, statistical differences in biomass, average thickness, roughness coefficient, and maximum thickness between strains were calculated using a two-way ANOVA with Sidak’s multiple comparison test in GraphPad PRISM (version 9.1.0).

For TMT, proteins with fold change (FC) > 1.2 were considered upregulated, and FC < 0.8 were considered downregulated. These cut-offs are widely used by other proteomic studies (34). A two-tailed Student’s *t*-test with Benjamini-Hochberg (BH) correction was performed in Proteome Discover to assess statistical significance. Finally, for MRM-hr, the results were analyzed on Skyline in MS-stats (version 4.1.2), an inbuilt R package (36), using a *t*-test with BH correction. For TMT and MRM-hr, a BH-corrected *P*-value (*q* value) < 0.05 was considered statistically significant (34).

## RESULTS

### Comparison of biofilm formation between SNP cluster I and SNP cluster II strains

**Fig 1:**
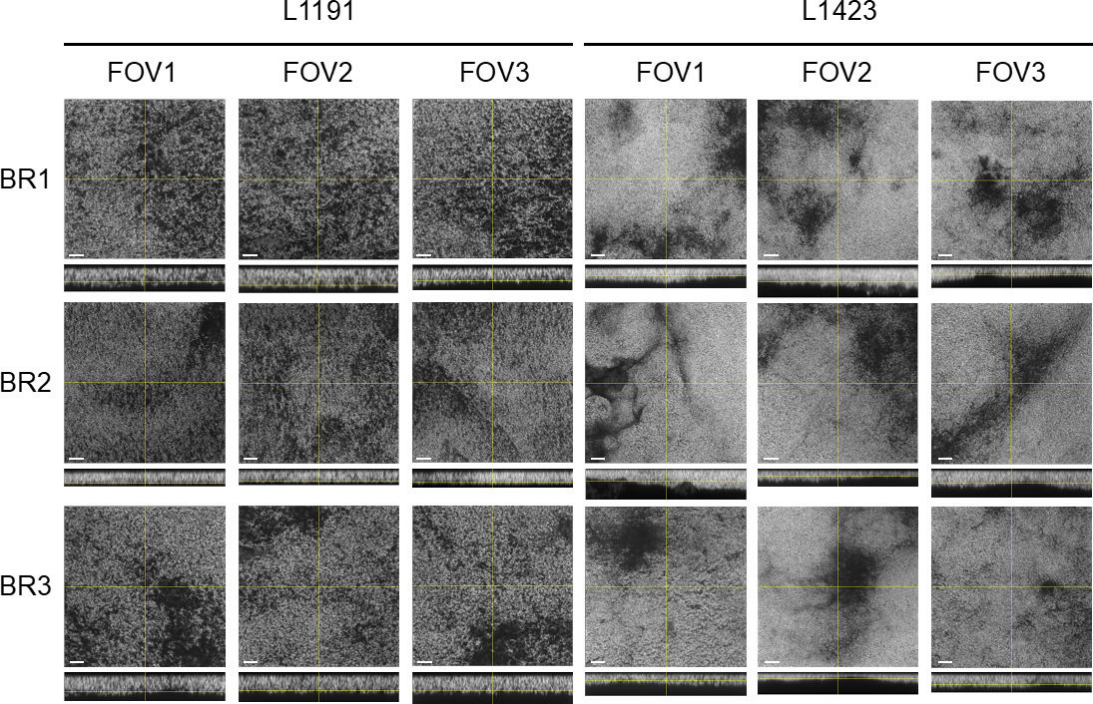
Confocal laser scanning microscopy (CLSM) micrographs of *B. pertussis* biofilms at 96 h. SNP cluster I strain, L1423, and SNP cluster II strain, L1191, were grown on glass cover slips and imaged at 96 h. Biofilms were stained with SYTO 9 fluorescent dye and visualized using CLSM. Z-stack images were taken at 0.42 µm intervals. The bar represents 10 µm distance; *xy* and *xz* representative focal planes are shown. Images are represented in original grayscale for easier visibility of contrast (39). BR, biological replicate; FOV, field of view.

**Fig 2:**
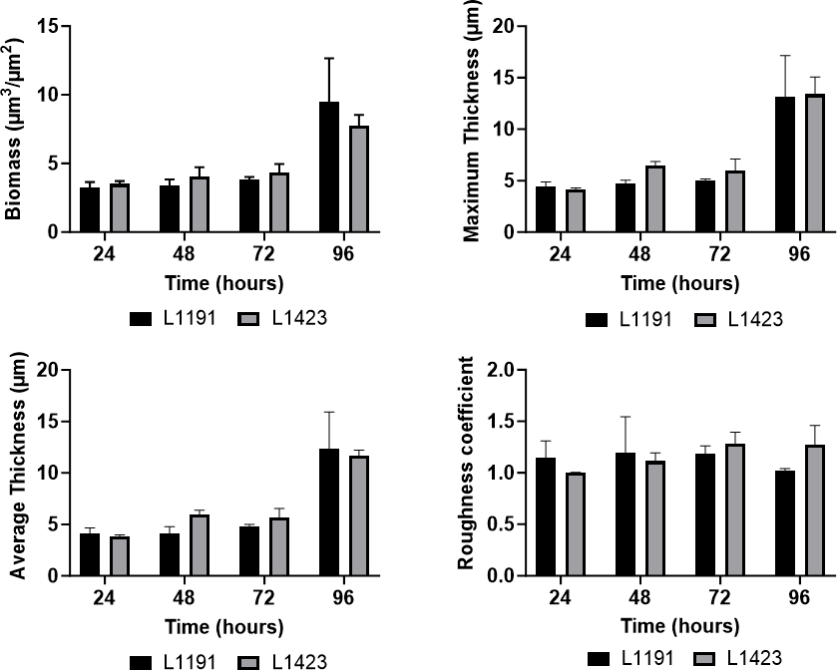
COMSTAT2 analysis of *B. pertussis* CLSM biofilm images. SNP cluster I strain, L1423, and SNP cluster II strain, L1191, were grown on glass cover slips and imaged at 24, 48, 72, and 96 h. Microscopy images were analyzed using the COMSTAT2 ImageJ plugin. Biomass, maximum thickness, average thickness, and roughness coefficient were calculated. The data are averages of three fields of view per biological replicate, and three biological replicates were performed per strain per time point. Two-way ANOVA was performed, but no statistical difference between L1423 and L1191 was observed. Error bars represent standard deviation.

Confocal laser scanning microscopy was performed to identify changes in biofilm formation between a cluster I strain, L1423, and a cluster II strain, L1191. We confirmed that *B. pertussis* formed mature biofilms at 96 h as previously reported (15, 20, 27). While the biofilm formation over time was similar between the two strains, there were clear visual differences observed between the L1423 and L1191 strains at 96 h (Fig. 1). The L1423 strain showed a denser structure compared to L1191. Using the COMSTAT2 plugin on ImageJ, biomass, average thickness, roughness coefficient, and maximum thickness were measured for each strain. Despite the visual differences in structure at 96 h, there were no significant differences in biofilm average thickness, maximum thickness, biomass, or roughness coefficient between the two strains at any time point (Fig. 2).

### Comparison of biofilm proteins between cluster I strain, L1423, and cluster II strain, L1191

**Fig 3:**
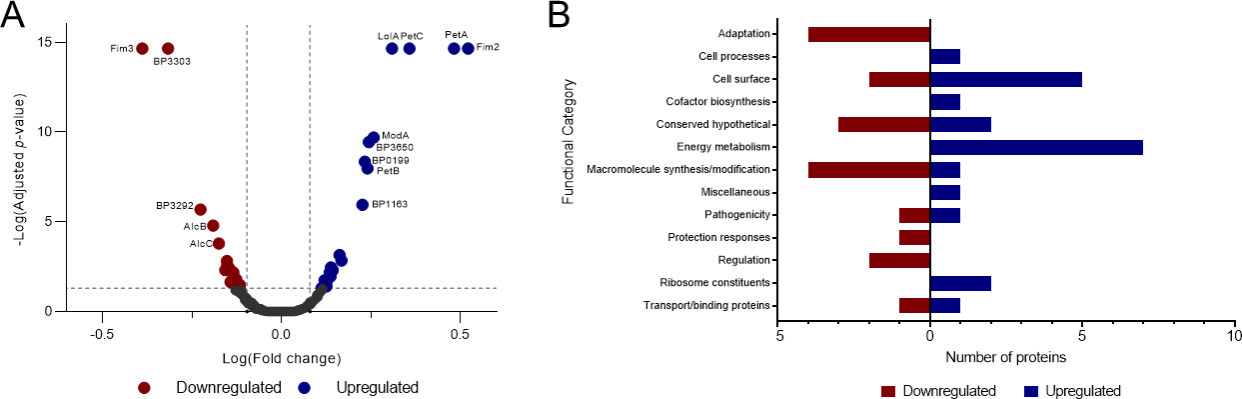
Proteins identified using TMT-MS between a representative *B. pertussis* SNP cluster I (L1423) and a SNP cluster II (L1191) strain. Proteins from a cluster I (L1423) and a cluster II (L1191) strain grown in biofilm conditions were extracted and measured by TMT-MS. (**A**) Volcano plot of proteins identified between the two strains. Proteins were deemed as upregulated with a fold change > 1.2, adjusted *P* < 0.05 and downregulated with a FC < 0.8, adjusted *P <* 0.05. Dashed gray lines mark 0.8 > FC > 1.2 and an adjusted *P* value = 0.05. Proteins with adjusted *P* < 0.001 are labeled on the plot. (**B**) Significantly differentially regulated proteins were grouped into functional categories based on Bart et al. (2). The number of proteins downregulated within each category is shown in red, and the number of upregulated proteins is shown in blue.

To identify the underlying proteins that may have led to the differences seen in the CLSM analysis, TMT-MS was performed on L1423 and L1191 biofilm-extracted proteins. There were 1,453 proteins identified, which is ~45% of the total known *B. pertussis* proteins (Table S2). A high proportion of proteins identified were predicted to be located in the cytoplasm (57%), while 14% of the proteins were located in the cytoplasmic membrane. The other proteins were distributed in the periplasmic space, outer membrane, and extracellular space at 4%, 3%, and 1%, respectively. The rest of the protein locations were unable to be predicted (19%). There were 40 proteins that were identified with significant (0.8 > FC > 1.2, adjusted *P* < 0.05) differences in abundance between the two strains (Fig. 3A). There were 18 proteins that were significantly downregulated and 22 significantly upregulated in L1423 compared to L1191 (Tables 2 and 3).

**TABLE 2:** Proteins downregulated in L1423 *B. pertussis* biofilm cells compared to L1191^*c*^

Locus	Gene	Product	Functional category	Fold change (L1423/L1191)	Adjusted *P-*value	Downregulation identified in L1423 vs L1191 planktonic^*a*^	Proteins differentially abundant in L1423 biofilm vs L1423 planktonic^*b*^
BP0437		LysR family transcriptional regulator	Regulation	0.764	0.0341	Not detected	Not detected
BP0521		Hypothetical protein BP0521	Conserved hypothetical	0.747	0.0163	No	Downregulated in biofilm
BP0782		Exported protein	Cell surface	0.720	0.0227	No	Not detected
BP1306	*arsC*	Arsenate reductase	Protection responses	0.704	0.0015	No	No difference
BP1364		Amino acid ABC transporter substrate-binding protein	Transport/binding proteins	0.761	0.0466	No	Downregulated in biofilm
BP1548		Nuclease/helicase	Macromolecule synthesis/ modification	0.723	0.0199	No	Not detected
BP1568	*fim3*	Serotype 3 fimbrial subunit	Pathogenicity	0.408	2.26E-15	Yes	Not detected
BP1616	*dps*	DNA-binding protein	Adaptation	0.711	0.0035	No	Downregulated in biofilm
BP2457	*alcB*	Alcaligin biosynthesis protein	Adaptation	0.644	1.62E-05	Not detected	Not detected
BP2458	*alcC*	Alcaligin biosynthesis protein	Adaptation	0.668	0.0002	No	Not detected
BP2520		LysR family transcriptional regulator	Regulation	0.734	0.0066	No	Not detected
BP2652		Exported protein	Cell surface	0.738	0.0163	No	No difference
BP3037		Hypothetical protein BP3037	Conserved hypothetical	0.705	0.0027	No	Downregulated in biofilm
BP3240	*uvrA2*	Excinuclease ABC subunit	Macromolecule synthesis/ modification	0.696	0.0049	No	Not detected
BP3292		Primosomal protein	Macromolecule synthesis/ modification	0.594	2.11E-06	No	Not detected
BP3303		ADP-dependent (S)-NAD(P)H-hydrate dehydratase	Conserved hypothetical	0.482	2.26E-15	Yes	Not detected
BP3486	*nusB*	Transcription antitermination protein NusB	Macromolecule synthesis/ modification	0.760	0.0442	No	Downregulated in biofilm
BP3530	*hupB*	DNA-binding protein HU-beta	Adaptation	0.714	0.0042	No	Downregulated in biofilm

^*a*^ Protein changes were compared to Luu et al.’s (13) study to identify proteins that were also downregulated in L1423 vs L1191 planktonic cells.

^*b*^ Protein changes were compared to Suyama et al.’s (27) study to identify proteins that were also differentially expressed in L1423 biofilms vs planktonic cells.

^*c*^ Not detected means the protein was not detected in the previous studies.

**TABLE 3:** Proteins upregulated in L1423 *B. pertussis* biofilm cells compared to L1191^*c*^

Locus	Gene	Product	Functional category	Fold change (L1423/L1191)	Adjusted *P-*value	Upregulation identified in L1423 vs L1191 planktonic^*a*^	Protein differentially abundant in L1423 biofilm vs L1423 planktonic^*b*^
BP0199		Hypothetical protein BP0199	Conserved hypothetical	1.737	1.06E-08	Not detected	Upregulated in biofilm
BP0275	*petC*	Cytochrome C1	Energy metabolism	2.275	2.26E-15	Not detected	No difference
BP0276	*petB*	Cytochrome B	Energy metabolism	1.707	4.57E-09	Not detected	Not detected
BP0277	*petA*	Ubiquinol-cytochrome C reductase iron-sulfur subunit	Energy metabolism	3.034	2.26E-15	No	Downregulated in biofilm
BP0386		Thioredoxin	Cofactor biosynthesis	1.384	0.0055	Not detected	Biofilm unique
BP0748	*rpmA*	50S ribosomal protein L27	Ribosome constituents	1.363	0.0066	Not detected	Downregulated in biofilm
BP0842	*nuoB*	NADH-quinone oxidoreductase subunit B	Energy metabolism	1.334	0.0229	No	No difference
BP0848	*nuoH*	NADH-quinone oxidoreductase subunit H	Energy metabolism	1.294	0.0489	No	Not detected
BP1119	*fim2*	Serotype 2 fimbrial subunit	Pathogenicity	3.319	2.26E-15	Yes	Downregulated in biofilm
BP1163		Short-chain dehydrogenase	Miscellaneous	1.682	1.11E-06	Not detected	No difference
BP1329		Malto-oligosyltrehalose trehalohydrolase	Macromolecule synthesis/modification	1.335	0.0399	Not detected	Not detected
BP1736		Exported protein	Cell surface	1.451	0.0007	No	Not detected
BP2140		Membrane protein	Cell surface	1.470	0.0014	Not detected	Not detected
BP2379	*hslO*	Hsp33 family chaperonin	Cell processes	1.369	0.0111	No	Not detected
BP2472	*lolA*	Outer-membrane lipoprotein carrier protein	Cell surface	2.034	2.26E-15	No	No difference
BP2924		Exported protein	Cell surface	1.389	0.0049	Yes	Upregulated in biofilm
BP3088		Exported protein	Conserved hypothetical	1.373	0.0035	No	Not detected
BP3095	*modA*	Molybdate ABC transporter substrate-binding protein ModA	Transport/binding proteins	1.807	2.02E-10	Yes	Downregulated in biofilm
BP3288	*atpD*	ATP synthase subunit beta	Energy metabolism	1.315	0.0400	No	Upregulated in biofilm
BP3440		Exported protein	Cell surface	1.319	0.0184	No	Biofilm unique
BP3615	*rplW*	50S ribosomal protein L23	Ribosome constituents	1.313	0.0413	No	Downregulated in biofilm
BP3650		Cytochrome C	Energy metabolism	1.754	3.62E-10	No	Downregulated in biofilm

^*a*^ Protein changes were also upregulated in L1423 vs L1191 planktonic cells from Luu et al.’s (13) study.

^*b*^ Protein changes were compared to Suyama et al.’s (27) study to identify proteins that were also differentially expressed in L1423 biofilms vs planktonic cells.

^*c*^ Not detected means the protein was not detected in the previous studies.

The largest change was seen in proteins belonging to the energy metabolism functional category (Fig. 3B). There were seven proteins upregulated in energy metabolism in L1423. These include four cytochrome proteins (PetABC and BP3650) as well as NADH-quinone oxidoreductase subunits (NuoBH) and ATP synthase (AtpD). There were only two proteins related to pathogenicity that were differentially regulated: Fim2 was upregulated in L1423, while Fim3 was upregulated in L1191 biofilm cells. Interestingly, two siderophore biosynthesis proteins, AlcBC, were downregulated in the L1423 biofilm cells. Proteins that were previously shown to have significant changes between the two strains in planktonic conditions (13), ModA and BP3303, were also seen to be both upregulated and downregulated in L1423, respectively. Two LysR transcriptional regulator proteins (BP0437 and BP2520) were downregulated as well as two DNA-binding proteins (HupB and Dps). Finally, there were five cell surface proteins that were upregulated in L1423 biofilm cells (Fig. 3B).

### Confirmation of protein abundance differences with MRM-hr

MRM-hr mass spectrometry was used to confirm the changes in abundance identified in the TMT-MS experiment above. Of the 40 proteins with significantly differential abundance between the strains, 9 proteins with suitable peptides were selected for confirmation with MRM-hr (Table 4; Table S1). Fim2 and Fim3 were excluded from the analysis as they were related to the strain serotype, which is already known (10). The upregulated proteins tested were PetC, BP3650, BP2924, AtpD, and RplW. The downregulated proteins tested were AlcC, NusB, BP1364, and BP2652. Three non-differentially abundant proteins, DnaK, ClpB, and RpsA, were selected as the control proteins for normalization (Table 4).

**TABLE 4:** Proteins tested with MRM-hr for confirmation of TMT-MS results

Locus	Gene	Product	Functional category	Fold change (TMT)	Adjusted *P-*value (TMT)
BP0275	*petC*	Cytochrome C1	Energy metabolism	2.275	2.26E-15
BP0950^*a*^	*rpsA*	30S ribosomal protein S1	Ribosome constituents	0.961	0.997
BP1198^*a*^	*clpB*	ATP-dependent protease, ATPase subunit	Macromolecule degradation	0.945	0.997
BP1364		Amino acid ABC transporter substrate-binding protein	Transport/binding proteins	0.761	0.047
BP2458	*alcC*	Alcaligin biosynthesis protein	Adaptation	0.668	1.60E-04
BP2499^*a*^	*dnaK*	Molecular chaperone DnaK	Cell processes	0.924	0.997
BP2652		Exported protein	Cell surface	0.738	0.016
BP2924		Exported protein	Cell surface	1.389	0.005
BP3288	*atpD*	ATP synthase subunit beta	Energy metabolism	1.315	0.040
BP3486	*nusB*	Transcription antitermination protein NusB	Macromolecule synthesis/ modification	0.760	0.044
BP3615	*rplW*	50S ribosomal protein L23	Ribosome constituents	1.313	0.041
BP3650		Cytochrome C	Energy metabolism	1.754	3.62E-10

^*a*^ Denotes selection as control proteins for normalization.

**Fig 4:**
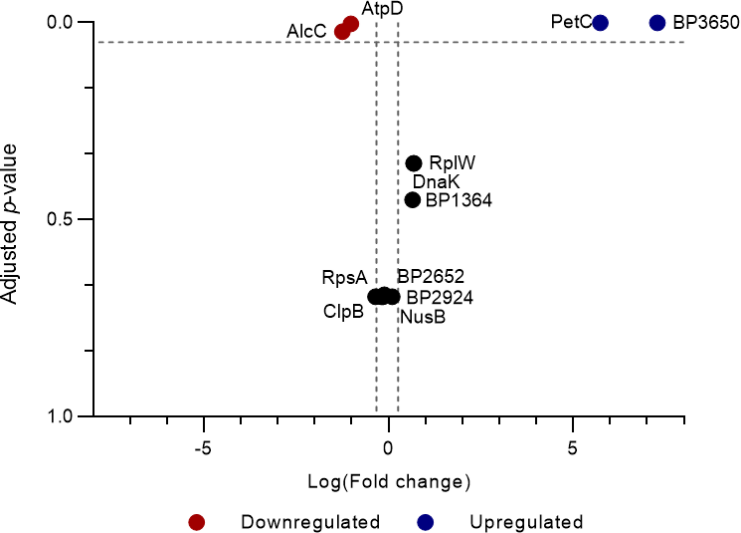
Confirmation of differentially abundant proteins identified in the TMT-MS using high-resolution multiple reaction monitoring**.** Volcano plot of nine proteins tested using MRM-hr. Three control proteins, DnaK, RpsA, and ClpB, were chosen based on the TMT-MS results for normalization. Proteins were deemed as upregulated with a fold change > 1.2, adjusted *P* < 0.05 and downregulated with a FC < 0.8, adjusted *P <* 0.05. Dashed gray lines mark 0.8 < FC > 1.2 and an adjusted *P* value = 0.05.

The MRM-hr experiment confirmed PetC and BP3650 to be upregulated in L1423, while AlcC was confirmed to be downregulated in L1423 (adjusted *P*-value < 0.05) (Table S3). AtpD was also seen to be downregulated in L1423, which was the opposite of the TMT result (Fig. 4).

**Fig 5:**
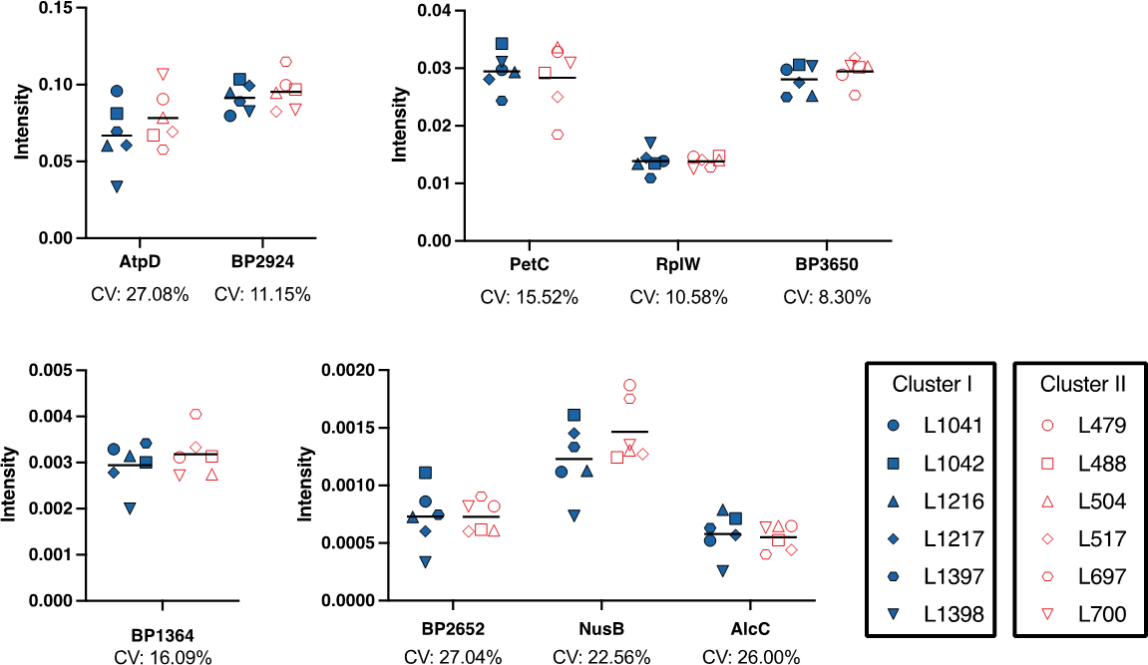
High-resolution multiple reaction monitoring measurement of TMT-MS proteins of six additional strains from SNP cluster I and SNP cluster II. Relative abundances of proteins from individual strains were measured by MRM-hr. Six additional *B. pertussis* strains from both clusters were tested for a subset of the differentiated proteins identified from the representative strains. The clusters are represented as different colors, and the strains as separate symbols. The protein intensity value (*y*-axis) is an average of all peptides measured for that protein for each strain. The bar indicates the average for the cluster. Coefficients of variation (CV) are indicated under the individual proteins. The proteins are grouped into separate graphs to allow differences in scale. No statistically significant differences were observed between SNP clusters I and II for all proteins measured (BH-corrected *P* < 0.05).

Furthermore, to confirm whether the differences identified are strain differences or cluster differences, MRM-hr was performed on six additional strains from each cluster, and the abundance of the same nine proteins mentioned above was measured. It was found that there were no significant differences in the abundance of these proteins between the clusters (Table S4). Interestingly, it was found that there was a high level of variability between strains (Fig. 5). The proteins with the highest variation were ATP synthase (AtpD) and exported protein (BP2652) (CV = 27.08% and 27.04%) with L700 (SNP cluster II strain) and L1042 (SNP cluster II strain) having the highest abundance of these proteins, respectively. Notably, SNP cluster I strain L1398 had the lowest abundance for AtpD, BP2652, and an alcaligin biosynthesis protein (AlcC). However, L1398 had the highest value for a ribosomal protein (RplW) and relatively high values for cytochrome proteins, BP3650 and PetC (Fig. 5).

**Fig 6:**
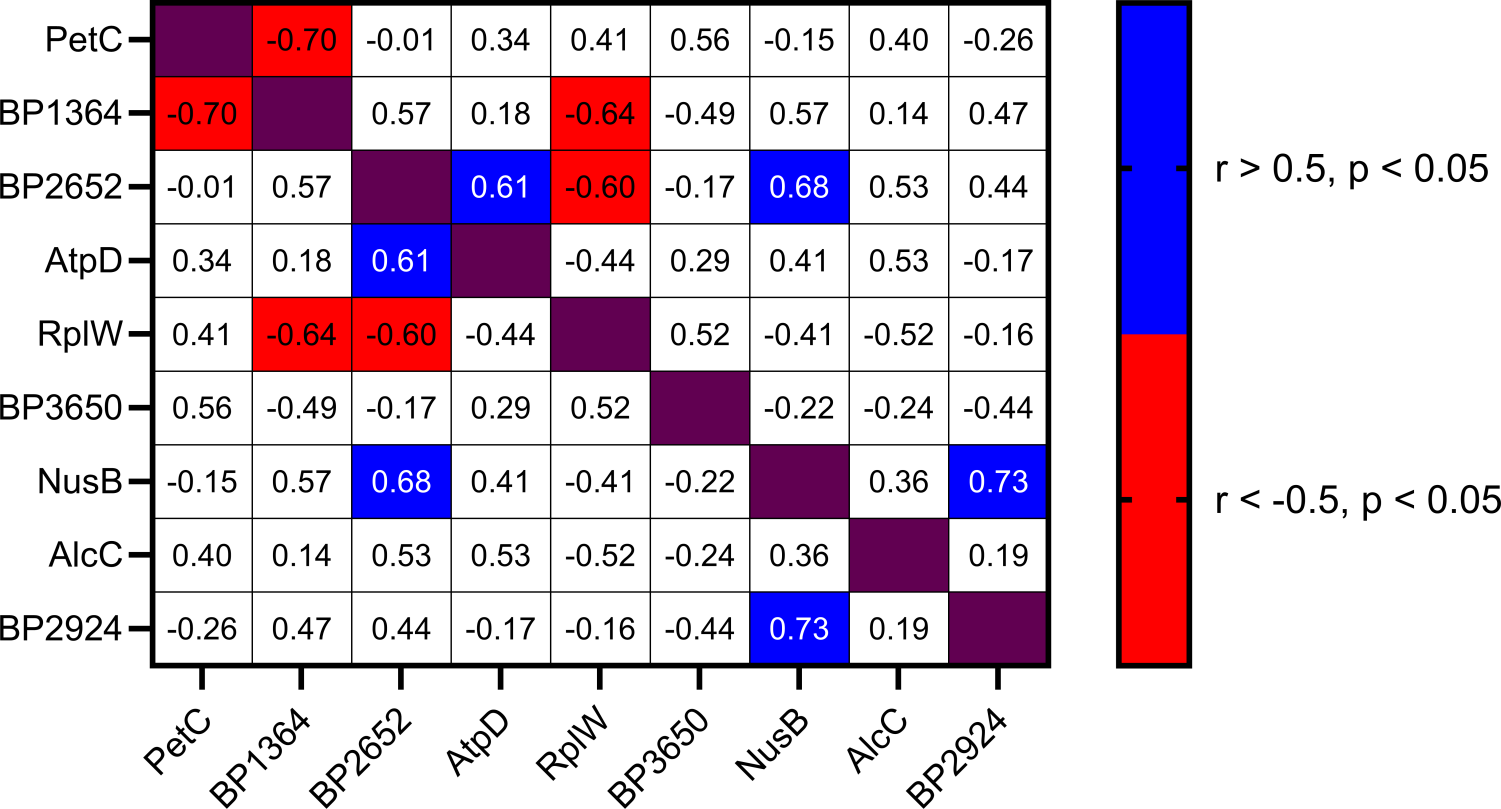
Pearson’s correlation matrix of significant correlations between protein abundance measured with MRM-hr**.** Protein average intensity was measured from 12 individual strains of *B. pertussis* from both SNP cluster I and SNP cluster II. Protein average intensity is the average of all peptides measured for that protein. Significant changes are shown in the matrix. Colors relate to negative (red) and positive (blue) correlations. Proteins were considered positively correlated if *r* > 0.5, *P* < 0.05 and negatively correlated if *r* < −0.5, *P* < 0.05.

There were correlations in abundance between particular proteins, suggesting that these proteins may be co-regulated. The abundance of BP2652 was positively correlated with NusB (Pearson’s correlation: *r* [10] = 0.677, *P* = 0.016) and AtpD (*r* [10] = 0.606, *P* = 0.037), while negatively correlated with RplW (*r* [10] = −0.559, *P* = 0.039) (Fig. 6). The abundance of NusB was also positively correlated with BP2924 (*r* [10] = 0.726, *P* = 0.008). Finally, BP1364 was negatively correlated with RplW (*r* [10] = −0.638, *P* = 0.026) and PetC (*r* [10] = −0.704, *P* = 0.011) (Fig. 6).

## DISCUSSION

Despite high vaccination coverage, there has been a resurgence in *B. pertussis* notifications globally (3, 40–43). It has been hypothesized that a contributing factor to the resurgence is related to the ability of *B. pertussis* to form biofilms (18, 44). We have previously investigated proteomic differences between planktonic and biofilm cells for a SNP cluster I strain (L1423), identifying >400 proteins differentially abundant in biofilms, including upregulation of toxins, downregulation of the type III secretion system, and a shift from cells utilizing the glyoxylate shunt in planktonic to the full TCA cycle in biofilms (27). Increased biofilm formation capability has also previously been hypothesized to increase respiratory tract colonization in clinical isolates (20). Also related to the resurgence is the expansion of SNP cluster I strains (*ptxP3* allele type) dominating over the previously dominant SNP cluster II strains (*ptxP1* allele type) (3, 24, 45, 46). Previous studies have examined differences between strains from these clusters in planktonic conditions but not biofilm (12, 13, 47). In this study, we compared cluster I and cluster II strains in biofilm formation and protein abundance to identify changes that may help explain the cluster I dominance. We found that the two SNP clusters had similar biofilm formation capabilities, but their biofilm structures were different.

First, a representative strain (L1423 and L1191) from each cluster was investigated. Both strains were chosen as they were the most well studied of each cluster and have both been sequenced and used for comparative infection in a mouse model and proteomic studies in planktonic conditions (10, 13). Additionally, L1423 was chosen as it belonged to SP13 and EL4. SP13 and EL4 are the predominant SNP profile and lineage, respectively, in cluster I that have been responsible for Australian epidemics between 2008–2017 (9, 48). EL4 is a large global lineage, and the most recent genotyping study published showed that EL4 has expanded in Australia from 2010 to 2019 and was the predominant epidemic lineage detected in 2019 (49). Thus, despite L1423 being isolated a decade ago, it is still epidemiologically relevant and representative of the most common lineage currently circulating in Australia and other developed countries using the ACV. For L1191, this strain was chosen as it was the most recent cluster II strain isolated during the same 2008–2010 epidemic. All other cluster II strains were isolated prior to 2008 and have not been detected in Australia since (49).

Both L1423 and L191 had consistent growth of biofilm and comparable levels of biomass at 96 h (Fig. 2). However, the cluster II strain was observed to have a more diffuse structure, while the cluster I strain had a denser biofilm structure (Fig. 1). Differences in *B. pertussis* mature biofilm structure have been reported between strains from two different regions (Argentina and USA) (20). It was found that the biofilms from strains isolated from Argentina made uniform layers of cells similar to those identified for both strains in this study, while the strains from the USA formed irregularly shaped aggregates (20). It should be noted that the structural differences identified between the Argentinian and USA *B. pertussis* strains (20) are more easily recognizable compared to those identified in this study. One possibility may be the use of SYTO 9 in this study, which binds all DNA, including extracellular DNA in the biofilm matrix. This may obscure biomass and biofilm structure differences from variations in biofilm extracellular DNA. Future studies using GFP-tagged strains can further delineate the biofilm structure and any extracellular DNA secreted by *B. pertussis*. Additionally, while the Cattelan et al. (20) study identified differences on the substratum level of bound biofilm (i.e., biofilms covering the entire surface vs clustered aggregates), the changes identified in this study relate to the density of the cells within the biofilm. The results presented in this study suggest that if the dominance of cluster I strains was related to biofilm, there are more subtle changes rather than overall biofilm formation capabilities that may contribute to the increased fitness previously observed (10).

This study explored differences in protein abundance between cluster I and cluster II strains in biofilm conditions to identify changes that may be related to the differences in structure. The most significant change between the two representative strains was upregulation of PetABC and BP3650 cytochrome proteins. PetABC is the cytochrome bc complex (complex III) that transfers electrons to cytochrome *c* (BP3650) and translocates protons across the membrane to create the proton motive force that powers ATP synthase (50). PetABC was found to be upregulated in iron-repressed conditions and in low oxygen conditions in other species (51–53). This suggests that the regulatory pathways that are induced in low iron or oxygen may be triggered in the cluster I strain. A low iron and oxygen environment could be related to a lower diffusion of the nutrients in the cluster I strain due to the denser biofilm structure identified in the confocal images.

Analysis of additional strains found that PetABC and BP3650 changes were strain specific to L1191 (Fig. 5). This may be explained by a genomic change that was observed specifically in the cluster II (L1191) strain. The sequences of L1423 and L1191 were aligned, and an insertion sequence (IS*481*) was found in the *petB* gene (nucleotide position 619920) in L1191 (Fig. S1). This insertion may disrupt the protein so that it is not produced correctly. The protein sequences were aligned to observe changes that may occur in the protein function. It was found that the insertion sequence led to a truncation (amino acid position 418) of the C-terminus, which includes a predicted transmembrane domain of the PetB protein that may affect its function as an electron transport chain complex (Fig. S2). This disruption may also lead to the changes observed in BP3650 abundance, as they are both linked as cytochrome proteins. While not a cluster-specific change, it is noteworthy that this change in abundance was not observed when the strains were compared planktonically (13). This suggests that the PetABC and BP3650 proteins may have a specific regulator that is only triggered in biofilm conditions for *B. pertussis*.

Our previous study had compared the same strains used in this study in planktonic conditions (13). Any overlap between the two studies would likely identify changes that are consistent between the two clusters, regardless of the condition (biofilm or planktonic). There were five proteins identified that had the same protein abundance trends between the studies (Tables 2 and 3). Of these, the downregulation of BP3303 and upregulation of ModA in the cluster I strain may be a result of genomic changes between the strains. Both genes encoding these proteins have non-synonymous SNPs, as mentioned in Luu et al*.* (13). BP3303 contains a SNP in L1423 at nucleotide position 699, causing a change from glycine to arginine. This SNP has been found in 19/22 of the cluster I strains sequenced from the Australian 2008–2012 epidemic (10). It is a hypothetical protein that requires further studies to elucidate its role. *modA* contains a SNP in L1191 at nucleotide position 29, causing a change from isoleucine to threonine. Both SNPs have been predicted to lead to reduced protein stability; however, the effect on protein functionality has not been experimentally determined (13). ModABC is required to transport molybdate, an essential metal in molybdoenzymes, and has been demonstrated to have a role in chronic lung infection and biofilm formation in *P. aeruginosa* (54, 55). Deletion of ModA reduced biofilm formation in *P. aeruginosa,* although the mechanism of how this occurs remains unknown (54). Whether and how ModABC plays a role in *B. pertussis* biofilm formation requires further investigation; however, in a previous study, *modB* has been shown to have increased abundance in *B. pertussis ptxP3* strains compared to *ptxP1* in sulfate-modulated conditions (24). These studies highlight a potential link between the protein abundance of ModABC and the dominance of the cluster I strains.

There were three additional proteins, Fim2, Fim3, and BP2924, identified in this study that reflected the results found in the planktonic comparison (13). Fim2 and BP2924 were upregulated, while Fim3 was downregulated in the cluster I strain. The cluster I strain is a Fim2 serotype, while the cluster II strain is a Fim3 serotype; therefore, these results confirm the expected changes in protein abundance (10). BP2924 is a hypothetical cell surface protein that was also found to be upregulated in the secretome of the same cluster I strain used in this study, although it could not be confirmed using MRM-hr (34). Furthermore, BP2924 was identified as transcriptionally upregulated in the Dutch *ptxP3* strains compared to the *ptxP1* strains (4). Therefore, BP2924 may be linked to a competitive advantage for the cluster I strains.

It has been well established that the current SNP cluster I (*ptxP3*) strains have an advantage over the previously dominant SNP cluster II (*ptxP1*) strains (4, 10, 11, 13). Due to the low genetic diversity in *B. pertussis* evolution, it is expected that additional strains from the cluster would reflect the changes identified in the representative strains (6, 8). Many of the changes identified in this study could not be confirmed using the MRM-hr method in the additional cluster strains due to a lack of suitable peptides. Of the 40 proteins identified as differentially regulated between the representative strains, only nine could be examined and were shown to have variable protein abundance for the additional strains included from each cluster (Fig. 5). Interestingly, it was seen that a collection of proteins displayed correlated trends in abundance between strains regardless of clusters (Fig. 6). This suggests that these proteins may be co-expressed or that the expression of one affects the other. Nevertheless, these results imply that the differences are likely strain-specific differences rather than cluster-specific differences and that it is unlikely that biofilm or biofilm protein abundance differences play a key role in the predominance of cluster I strains. It also suggests that SNP clusters do not predict biofilm behavior. Other potential explanations for the observed results are that strain-level differences within a cluster dominated over cluster-level trends and thus resulted in no statistically significant cluster-level differences. However, since the strains chosen for this study were from our extensive collection of clinical isolates and were based on our phylogenetic analysis (7, 9, 48), it is unlikely that the strains chosen were not representative of the clusters as an explanation for why no cluster-level differences were detected.

Previous studies have shown that the variability in gene expression between previously circulating and currently circulating strains is very small when considering the total number of coding genes (4, 13, 14, 24). With the exclusion of Fim2 and Fim3, there were an additional 29 proteins that were identified in the representative strains with differential abundance that were to be confirmed. These additional 29 proteins may still be cluster-specific changes and would require further investigation. Included within these proteins was a downregulation of two LysR transcriptional regulators and two DNA-binding proteins, HupB and Dps, in the cluster I strain. There is little research in the regulatory role of these proteins in *B. pertussis,* and studies have shown that there are regulators that control the expression of a wide range of *B. pertussis* genes and proteins (56–61). It is possible that these regulators are connected to the difference in biofilm structure and behavior between L1423 and L1191. Cluster I strains have been linked to increased disease severity (62). Future studies incorporating patient data such as vaccination status, disease severity, and age may identify relationships between biofilm protein abundance and clinical presentation. This study grew *B. pertussis* biofilms on tissue culture treated polystyrene plates. The use of different surfaces for bacterial attachment and biofilm can influence overall biofilm development and the set of proteins expressed such as for adhesion. Furthermore, previous proteomic studies identified greater differences in planktonic-secreted proteins between L1423 and L1191 than whole cell differences (12, 13); however, this study only examined proteins found within biofilm cells, and biofilm-secreted proteins were not investigated. Thus, it is possible that differences in biofilm-secreted proteins may also play a role in the dominance of cluster I strains. This study also only examined the mature biofilm structure and biofilm protein abundance at one time point (96 h), and there may be further biofilm differences at other time points between the clusters. It should also be noted that this study only investigated the abundance of canonical proteins annotated in the *B. pertussis* genomes used in the search database, and there may be differences in other non-canonical proteins or proteoforms in biofilms. For instance, a previous proteomic study identified 54 serine/threonine/tyrosine phosphorylation sites in *B. pertussis,* and differential phosphorylation of these sites between SNP cluster I and II strains may affect protein function and strain fitness (63).

In conclusion, this study did not identify differences in the amount of biofilm biomass produced between representative strains from cluster I and cluster II, suggesting that biofilm formation capability may not be a contributing factor to the fitness of the currently circulating cluster I strains over cluster II strains. However, further studies using a large number of strains from each cluster would be required to confirm the conclusion, given the large variations at the proteome level between strains within a cluster were detected. This study did identify differences in mature biofilm structure between the representative strains, which warrants further investigation. Understanding the underlying proteins that trigger these differences in biofilm behavior may provide insight into the biofilm formation process of *B. pertussis*. Differential abundance of BP3303, ModA, and BP2924 found in other studies (4, 13, 24) was confirmed to have the same trend in biofilm conditions between the cluster I and cluster II strains. The replicability of these findings suggests a potentially important role of these proteins in the dominance of cluster I strains. Finally, this study highlights that proteomic changes in biofilm conditions between clusters are compounded by strain-specific differences, underscoring the proteomic diversity of the *B. pertussis* population and contrasting the genetic homogeneity of the species (6, 8).

## Data Availability

The mass spectrometry proteomics data have been deposited to the ProteomeXchange Consortium via the PRIDE (64) partner repository with the data set identifier PXD046513.

## SUPPLEMENTAL MATERIAL

Supplemental figures spectrum.01715-25-s0001.docx Fig. S1 and S2.

Supplemental tables spectrum.01715-25-s0002.xlsx Tables S1 to S4.

ASM does not own the copyrights to Supplemental Material that may be linked to, or accessed through, an article. The authors have granted ASM a non-exclusive, world-wide license to publish the Supplemental Material files. Please contact the corresponding author directly for reuse.
