# Supplementary material for: Proteomic comparison of epidemic Australian Bordetella pertussis biofilm cells
Source: Microbiol Spectr. 2025 Sep 30;13(11):e01715-25. doi: 10.1128/spectrum.01715-25 (PMC12584666; doi:10.1128/spectrum.01715-25)
Supplement: Supplemental figures — Fig. S1 and S2. [file spectrum.01715-25-s0001.docx]

**Supplementary figures**


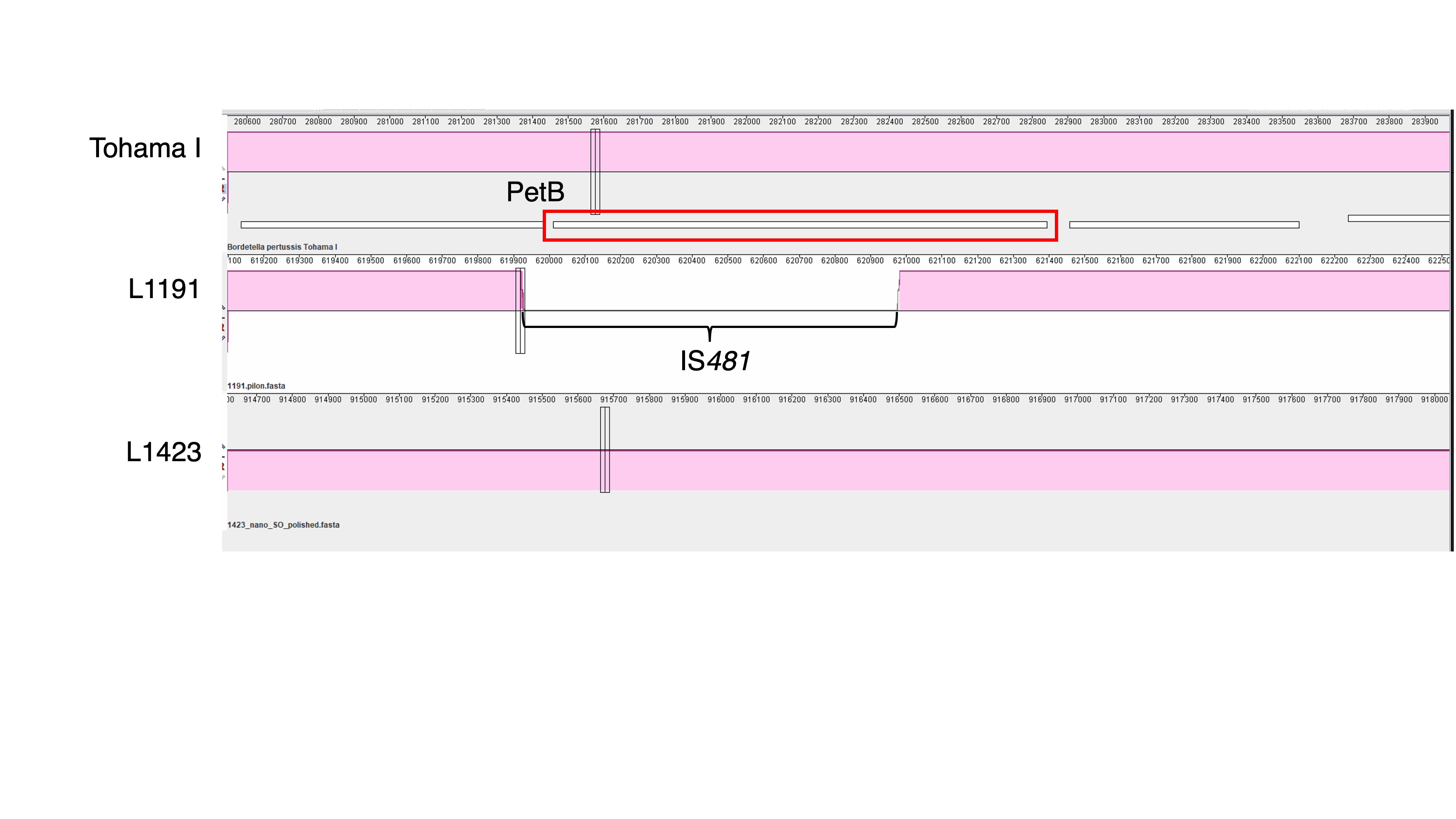
**Supplementary Figure S1 Comparison of L1191 and L1423 genome sequences**. ProgressiveMauve was used to align the sequences of *B. pertussis* genomes. L1423 and L1191 genomes were mapped against the Tohama I reference strain. The *petB* region is highlighted in red. The IS*481* disruption in the L1191 strain is marked. The black marker signifies the beginning of the IS disruption.

tr|Q7W081|Q7W081_BORPE MAGEKTVETTGLLGWLDQRFPVTSTWKAHLSEYYAPKNFNFWYFFGSLALLVLVIQIVTG 60

PetB_L1423 MAGEKTVETTGLLGWLDQRFPVTSTWKAHLSEYYAPKNFNFWYFFGSLALLVLVIQIVTG 60

PetB_1191 MAGEKTVETTGLLGWLDQRFPVTSTWKAHLSEYYAPKNFNFWYFFGSLALLVLVIQIVTG 60

************************************************************

tr|Q7W081|Q7W081_BORPE IFLVMHYKPDAERAFQSVEYIMREVPWGWLVRYMHSTGASMFFVVVYLHMLRGLFYGSYR 120

PetB_L1423 IFLVMHYKPDAERAFQSVEYIMREVPWGWLVRYMHSTGASMFFVVVYLHMLRGLFYGSYR 120

PetB_1191 IFLVMHYKPDAERAFQSVEYIMREVPWGWLVRYMHSTGASMFFVVVYLHMLRGLFYGSYR 120

************************************************************

tr|Q7W081|Q7W081_BORPE KPRELVWIFGVAIFLCLMGEAFFGYLLPWGQMSYWGAQVIVNLFSAIPFIGPELSIWIRG 180

PetB_L1423 KPRELVWIFGVAIFLCLMGEAFFGYLLPWGQMSYWGAQVIVNLFSAIPFIGPELSIWIRG 180

PetB_1191 KPRELVWIFGVAIFLCLMGEAFFGYLLPWGQMSYWGAQVIVNLFSAIPFIGPELSIWIRG 180

************************************************************

tr|Q7W081|Q7W081_BORPE DYVVSDATLNRFFSFHVIAIPLVLIGLVAAHLVALHEVGSNNPDGVEIKKGPKDKYGRPK 240

PetB_L1423 DYVVSDATLNRFFSFHVIAIPLVLIGLVAAHLVALHEVGSNNPDGVEIKKGPKDKYGRPK 240

PetB_1191 DYVVSDATLNRFFSFHVIAIPLVLIGLVAAHLVALHEVGSNNPDGVEIKKGPKDKYGRPK 240

************************************************************

tr|Q7W081|Q7W081_BORPE DGIPFHPFYTVHDIMGVAGFLIIFAAIVFFGPEMGGYFLEYNNFLPADPLKTPPHIAPVW 300

PetB_L1423 DGIPFHPFYTVHDIMGVAGFLIIFAAIVFFGPEMGGYFLEYNNFLPADPLKTPPHIAPVW 300

PetB_1191 DGIPFHPFYTVHDIMGVAGFLIIFAAIVFFGPEMGGYFLEYNNFLPADPLKTPPHIAPVW 300

************************************************************

tr|Q7W081|Q7W081_BORPE YFTPFYSMLRATTDVFTWVLAGAAILGAIALLLRAKGAMRIVAPVILVVVAVLLRTIDAK 360

PetB_L1423 YFTPFYSMLRATTDVFTWVLAGAAILGAIALLLRAKGAMRIVAPVILVVVAVLLRTIDAK 360

PetB_1191 YFTPFYSMLRATTDVFTWVLAGAAILGAIALLLRAKGAMRIVAPVILVVVAVLLRTIDAK 360

************************************************************

tr|Q7W081|Q7W081_BORPE FWGVVAMGGAVVILFFLPWLDHSPVKSIRYRPTWHKWLYGIFIVNFLVLGYLGTQPPSDA 420

PetB_L1423 FWGVVAMGGAVVILFFLPWLDHSPVKSIRYRPTWHKWLYGIFIVNFLVLGYLGTQPPSDA 420

PetB_1191 FWGVVAMGGAVVILFFLPWLDHSPVKSIRYRPTWHKWLYGIFIVNFLVLGYLGTQPPSCE 420

**********************************************************

tr|Q7W081|Q7W081_BORPE FNLTSQIGTLIYLGFFFLMPVWSRLGTFKPVPDRVTFHAH----- 460

PetB_L1423 FNLTSQIGTLIYLGFFFLMPVWSRLGTFKPVPDRVTFHAH----- 460

PetB_1191 DSIGCMHGSSE-----------PDLRNWKSPPPQFTQGARPDEHP 454

.: . *: * .:* * :.* *:

Supplementary Figure S2 Alignment of L1423 and L1191 PetB protein sequence. Translated sequences of the *petB* gene of L1423 and L1191 and the sequences were aligned on UniProt using the Clustal O (v 1.2.4) program. The sequences were aligned against the Tohama I PetB reference (tr|Q7W081|Q7W081_BORPE) protein sequence. Transmembrane domains are indicated in yellow. Identical sequences are marked with (*).
